# Mesoporous Co_3_O_4_@CdS nanorods as anode for high-performance lithium ion batteries with improved lithium storage capacity and cycle life†

**DOI:** 10.1039/d4ra01028k

**Published:** 2024-04-15

**Authors:** Hamza Waleed, Haroon Ur Rasheed, Faisal Faiz, Amina Zafar, Saqib Javed, Yanguo Liu, Shafqat Karim, Hongyu Sun, Yasir Faiz, Shafqat Hussain, Atia Khalid, Yanlong Yu, Amjad Nisar, Mashkoor Ahmad

**Affiliations:** a Nanomaterials Research Group, Physics Division, PINSTECH Islamabad 44000 Pakistan mashkoorahmad2003@yahoo.com chempk@gmail.com; b College of Electronics and Information Engineering, Shenzhen University Shenzhen PR China; c Central Analytical Facility Division, PINSTECH Islamabad 44000 Pakistan; d Theoretical Physics Division, PINSTECH Islamabad 44000 Pakistan; e School of Resources and Materials, Northeastern University at Qinhuangdao Qinhuangdao 066004 PR China; f Chemistry Division, PINSTECH Islamabad 44000 Pakistan; g School of Materials Science and Engineering, Tsinghua University Beijing China; h College of Chemistry and Chemical Engineering, Northeast Petroleum University Daqing 163318 PR China ylyu66@163.com; i Department of Physics, Faculty of Basic & Applied Sciences, IIU Islamabad 44000 Pakistan

## Abstract

Transition metal oxides based anodes are facing crucial problems of capacity fading at long cycles and high rates due to electrode degradations. In this prospective, an effective strategy is employed to develop advanced electrode materials for lithium-ion batteries (LIBs). In the present work, a mesoporous Co_3_O_4_@CdS hybrid sructure is developed and investigated as anode for LiBs. The hybrid structure owning porous nature and large specific surface area, provides an opportunity to boost the lithium storage capabilities of Co_3_O_4_ nanorods. The Co_3_O_4_@CdS electrode delivers an initial discharge capacity of 1292 mA h g^−1^ at 0.1C and a very stable reversible capacity of 760 mA h g^−1^ over 200 cycles with a capacity retention rate of 92.7%. In addition, the electrode exhibits excellent cyclic stability even after 800 cycles and good rate performance as compared to previously reported electrodes. Moreover, density functional theory (DFT) and electrochemical impedance spectroscopy (EIS) confirm the enhanced kinetics of the Co_3_O_4_@CdS electrode. The efficient performance of the electrode may be due to the increased surface reactivity, abundant active sites/interfaces for rapid Li^+^ ion diffusion and the synergy between Co_3_O_4_ and CdS NPs. This work demonstrates that Co_3_O_4_@CdS hybrid structures have great potential for high performance batteries.

## Abstract 2

Mesoporous Co_3_O_4_@CdS nanorods as anode for lithium-ion battery demonstrates enhanced lithium storage performance as compared to pristine Co_3_O_4_ structure due to the synergy between Co_3_O_4_ and CdS structures.

## Graphical abstract

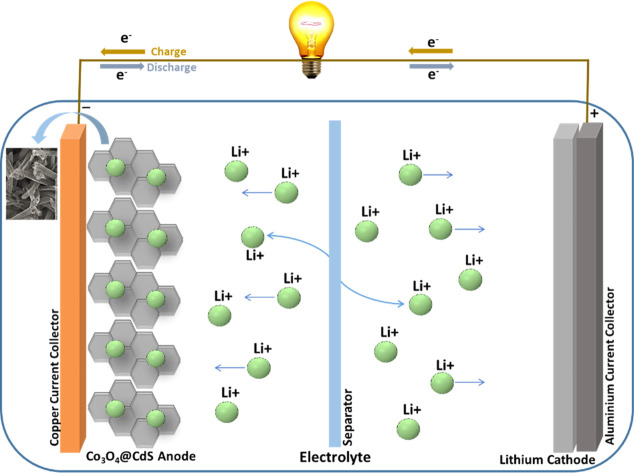

## 1 Introduction

With the rapid increase in the global energy crisis and environmental pollution, researchers directed their research activities to explore effective ways for store energy. Currently, nations around the world are working hard to produce clean, sustainable, and renewable energy sources including solar, wind, and ocean power. However, these sources need energy storage systems to regulate energy production. So far various energy storage devices such as batteries, supercapacitors and fuel cell *etc.* have been fabricated to store energy from renewable sources. Among these devices, rechargeable lithium-ion batteries (LIBs) is crucial to meet future needs for industries from personal devices to automobiles.^1^ However, durability and energy densities of current LIBs are restricted by electrode material.^2^ Therefore, it remains a great challenge to develop advanced electrode materials for LIBs that exhibits improved cycling stability and larger specific capacities. The current progress in the field of energy storage materials provides more innovative solutions to resolve the energy storage issues.

So far, various transition metal oxides (TMOs) such as MnO_2_,^3^ V_2_O_5_,^4^ ZnO,^5^ WO_3_,^6^ Co_3_O_4_,^7^*etc.* have been exploited as the anode materials for LIBs. Among these, cobalt oxide (Co_3_O_4_) is regarded as one of the most promising anode materials due to its high theoretical capacity (∼890 mA h g^−1^), environmental friendliness, and superior electrochemical properties. However, its poor cyclic stability, irreversible capacity loss and slow kinetics of Li-ion and electron transport hinder its practical application. In order to overcome these drawbacks, many attempts have been made such as the use of conductive polymers,^8^ doping with transition metals,^9^ and making composites.^10^ On the other hand, recent work on metal sulphides has drawn considerable attention for being the most promising electrode materials for LIBs due to their high electrical conductivity, thermal durability and rich redox chemistry than their metal oxides equivalents. To explore novel materials for energy storage devices, metal sulphides provide better option due to its outstanding features. Among various metal sulphides, cadmium sulfide (CdS) has received less attention as an electrode material for LIBs. Therefore, in order to explore the energy storage features of Co_3_O_4_ and CdS in a single system, their combination is found to be a good approach for the development of novel electrode material. Such hybrid systems are very appealing for energy storage applications due to their porous nature and ability to absorb guest species such as lithium ion on their surfaces and in the pore spaces. Up till now, according to our knowledge, there have been no reports found on the investigation of Co_3_O_4_@CdS nanorods as an electrode material for LIBs. For example, D. S. Patil, *et al.*, have reported the use of the core–shell structure of Co_3_O_4_@CdS for high-performance supercapacitors,^11^ F. Q. Liu, *et al.*, prepared Co_3_O_4_/CdS photoelectrode for photoelectrochemical cathodic protection in the dark^12^ and Z. Qin *et al.*, have developed Co_3_O_4_/CdS p–n heterojunction for enhancing photocatalytic hydrogen production.^13^ C. Jiang *et al.*, prepared Co_3_O_4_@CdS Hollow Spheres by the template-removal method with the assistance of the ZIF-67 material and obtained high phenol and dye photodegradation activity.^14^

In this work, mesoporous Co_3_O_4_@CdS nanorods are developed and investigated as an anode material for LIBs. The hybrid structure shows enhanced physical and chemical properties superior to a single counterpart. The porous nature, large specific surface area and excellent kinetics of the synthesized nanorods leads to rapid lithium storage. The developed electrode exhibits enhanced cycling stability and high-rate capability as compared to pristine Co_3_O_4_ nanorods. Therefore, Co_3_O_4_@CdS structure can be considered suitable candidate as an anode material for LIBs.

## 2 Experimental

The experimental detail is given in the (ESI).†

## 3 Results and discussion

### 3.1. Morphological, structural and compositional analysis

**Fig. 1:**
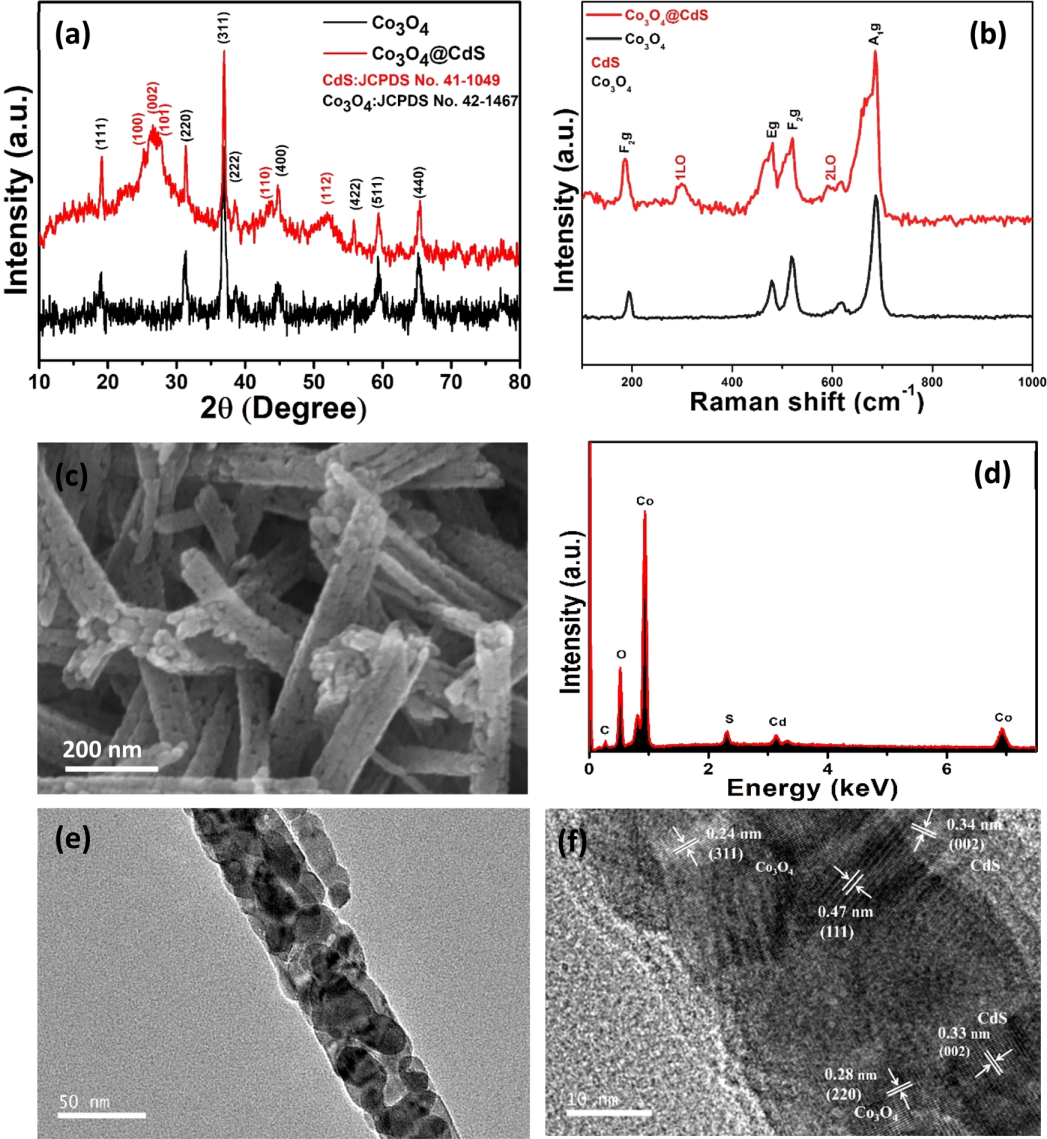
(a) XRD patterns of pure Co_3_O_4_ and Co_3_O_4_@CdS structures. (b) Raman spectra of Co_3_O_4_ and Co_3_O_4_@CdS NPs structures prepared through deposition by modified SILAR method. (c) FE-SEM image of Co_3_O_4_@CdS structures; (d) corresponding EDX spectrum of Co_3_O_4_@CdS structures (e) TEM image of Co_3_O_4_@CdS structure (f) HRTEM of Co_3_O_4_@CdS structure.

XRD analysis was performed in order to investigate the phase and crystallinity of the as-prepared Co_3_O_4_ and Co_3_O_4_@CdS structures as shown in Fig. 1(a). In pristine Co_3_O_4_ pattern, the diffraction peaks at 19°, 31.3°, 37°, 38.5° and 44.7° are assigned to (111), (220), (311), (222) and (400) planes of Co_3_O_4_ according to JCPDS # 00-042-1467.^15^ The diffraction pattern of Co_3_O_4_@CdS structure shows the additional peaks located at 25.2°, 26.8°, 27.6°, 43.7°and 52.1° attributed to the (100), (002), (101), (110) and (112) planes of CdS according to (JCPDS # 00-041-1049) and confirms the deposition of CdS.^16^ No other peaks related to impurities are observed, demonstrating the purity of both samples.

In order to understand the vibrational behaviour of the prepared structures, Raman spectra of Co_3_O_4_ and Co_3_O_4_@CdS structures were recorded in the range of (100–1000) cm^−1^ as shown in Fig. 1(b). In Co_3_O_4_ spectrum, the bands located at 185, 478, 520, and 686 cm^−1^are associated to F_2g_, E_g_, F_2g_ and A_1g_ modes respectively.^17^ In Co_3_O_4_@CdS spectrum, besides Co_3_O_4_ bands, two characteristic bands of CdS at 300 cm^−1^ and 600 cm^−1^ were also recorded which are attributed to the fundamental longitudinal optical phonon (1LO) and its overtone longitudinal phonon (2LO) respectively.^18^ In comparison with Co_3_O_4_ spectrum, a small peak located at 413 cm^−1^ is also observed, which ascribed to the Co–S bond formation on the surface of Co_3_O_4_@CdS nanorods.^19^ With the close observation, a slight shift was also observed in the position of 1LO and 2LO modes which is due to the shape of the nanostructures.^20^ In addition, the composite spectrum exhibits broadening associated with the slight changes in the crystalline structure of pristine Co_3_O_4_ as mentioned by previous reports.^21^

To examine the morphology of the as-prepared Co_3_O_4_@CdS structure, SEM was performed. Fig. 1(c) shows the SEM images of Co_3_O_4_@CdS nanorods. As observed from the images, the Co_3_O_4_ nanorods are composed of highly dense CdS NPs interconnected with one another in uniformly ordered arrays. For comparison, the SEM images of the pristine Co_3_O_4_ nanorods and Co_3_O_4_@CdS nanorods are also recorded as shown in Fig. S1 (ESI), S2(a and b)† respectively. The surface of the nanorods appear to be uniform and smooth. Fig. 1(d) represents the corresponding EDX spectrum of Co_3_O_4_@CdS which consists of Cd, S, Co, and O peaks, confirm the formation of Co_3_O_4_@CdS nanorods. The existence of Cd and S peaks further confirms the successful deposition of CdS NPs. In order to clarify the detail structure and elemental distribution of the prepared composite, EDX elemental mapping are performed. Fig. S3(a–f)† displays the STEM images of Co_3_O_4_@CdS structure and a corresponding elemental mapping of Co, O, Cd and S respectively. The presence of Cd and S confirms the deposition of CdS and no other impurities formed other than Co_3_O_4_ and CdS. The detailed structural analysis was conducted *via* TEM and HRTEM. Fig. 1(e) exhibits a low magnification TEM image of Co_3_O_4_@CdS structure, demonstrating the deposition of CdS NPs on the whole surface of Co_3_O_4_ nanorods. Fig. 1(f) displays the HRTEM analysis of the Co_3_O_4_@CdS structure and exhibits a polycrystalline nature. The well resolved lattice fringes of 0.24, 0.47 are observed corresponding to (311) and (111) planes of Co_3_O_4_ nanorods. The lattice fringe of 0.33 nm belongs to (002) plane of CdS is also identified.

FTIR analysis was carried out to examine and investigate the structural molecular changes and presence of different functional groups in as-prepared Co_3_O_4_@CdS structures. Fig. S4 (ESI)† illustrates the FTIR spectra of the Co_3_O_4_ and Co_3_O_4_@CdS structures. The bands sited at 555 cm^−1^ and 657 cm^−1^ reveals the stretching vibrations which show the presence of Co–O bonding. The band located at 1039 cm^−1^ is illustrating the presence of Cd–S interaction and manifests the formation of the Co_3_O_4_@CdS composite. A sharp peak sited at 1458 cm^−1^ represents the bending vibrations of monodispersed Co_3_O_4_ structure. Furthermore, the significant peaks sited at 2851 cm^−1^ and 2918 cm^−1^ shows the symmetric and asymmetric stretching modes of –CH_2_ which comprises from HMT which plays a significant role as a nucleation controlled reagent and thus, favouring the surface modification of Co_3_O_4_ nanorods.

**Fig. 2:**
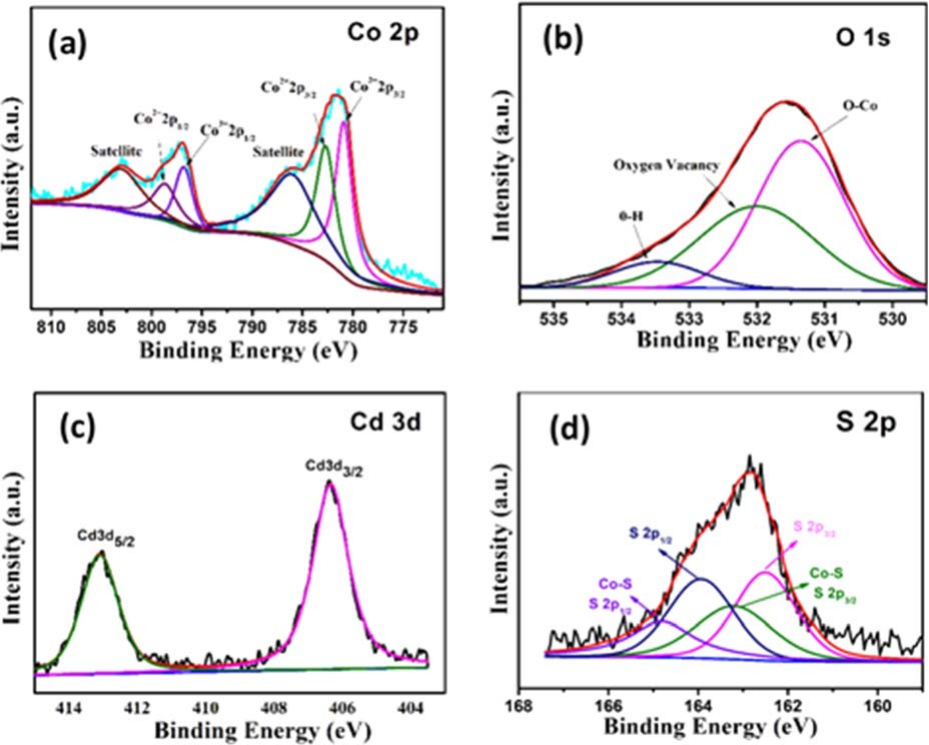
High resolution XPS spectrum of (a) Co 2p (b) O 1s (c) Cd 3d and (d) S 2p peak.

The surface of the nanostructures plays an important role in the electrochemical performance of the material. Therefore, XPS of Co_3_O_4_@CdS is measured to examine the surface-localized information of the elements. Fig. 2(a) shows the deconvoluted XPS spectrum of Co 2p, which consists of two regions at 796.7 eV and 780.9 eV corresponding to Co 2p_1/2_ and Co 2p_3/2_ states respectively. In the Co 2p_3/2_ region, two coexisting peaks at 781.1 eV and 783.0 eV can be seen which confirms the Co^3+^ and Co^2+^ states. Moreover, Co 2p_1/2_ region also composed of two peaks at 797.3 eV and 798.3 eV related to Co^3+^ and Co^2+^ states. Furthermore, two satellites peaks at 786.7 eV and 803.3 eV corresponding to Co 2p_3/2_ and Co 2p_1/2_ regions, reconfirms the formation of Co^2+^ and Co^3+^ states and agrees well with the previous report.^22^ For comparison, XPS spectra of Co 2p region of Co_3_O_4_ structures is also recorded as shown in Fig. S5 (ESI).† The area ratio of Co^3+^ to Co^2+^ of both structures are calculated. As observed the relative area ratio of Co_3_O_4_@CdS (1.14) is significantly higher as compare to Co_3_O_4_ (1.07) confirming that the Co^3+^ states are more exposed on the surface of Co_3_O_4_@CdS structure. It is already reported that the nanostructure with dominant Co^3+^ sites exhibit superior electrochemical performance.^23–25^ Thus Co_3_O_4_@CdS structure with dominant Co^3+^ active sites are considering more suitable for energy storage applications. Fig. 2(b) depicts the high-resolution spectrum of O 1s. The spectrum fitted into three peaks located at 531.4, 532.1 and 533.5 eV corresponding to the O–Co bond, oxygen vacancy defects and O–H species due to the surface absorption of water. The existence of Cd^2+^ is confirmed by the high-resolution spectrum of Cd 3d peak as shown in Fig. 2(c). The spectrum reveals two binding energy peaks at 406.41 and 411.14 eV correspond to the electronic states of Cd 3d_5/2_ and Cd 3d_3/2_, respectively. The energy difference between two peaks is 4.73 eV, which agrees well with the reported study.^26^Fig. 2(d) demonstrates the spectrum of S 2p peaks which comprised two major spin–orbit peaks located at 162.3 eV and 163.9 eV corresponding to the S 2p_3/2_ and S 2p_1/2_ states respectively. In addition, two peaks at 163.2 eV and 164.7 eV corresponding to the S 2p_3/2_ and S 2p_1/2_ states can be seen which ascribed to the formation of Co–S bond on the surface of Co_3_O_4_@CdS nanorods.

Fig. S6 (ESI)† shows the N_2_ adsorption–desorption isotherm of Co_3_O_4_ and Co_3_O_4_@CdS structures determined at 77 K. It can be observed that the isotherm of Co_3_O_4_@CdS structures, presents a broad hysteresis loop as compared to pristine Co_3_O_4_ structure. The corresponding surface area of Co_3_O_4_ and Co_3_O_4_@CdS structures was calculated to be 43 m^2^ g^−1^ and 81 m^2^ g^−1^ respectively. The increased surface area of the hybrid structure is considered due to the CdS NPs and the porous nature of Co_3_O_4_@CdS structures. Moreover, the pore size of Co_3_O_4_@CdS structures, based on Barrett–Joyner–Halenda (BJH) Model, is also measured by the pore size distribution curve as illustrated in the inset of Fig. S6 (ESI).† The calculated average pore size is ∼11 nm. The interconnected primary nanoparticles and the void spaces within a single nanorod is the primary sources for the formation of mesopores. It is well-known that the porous structure with a high electroactive surface area, plays a vital role in the electrochemical processes.^27^ Thus, the provision of such good mesoporous surface, accompanying abundant active sites, proves to be beneficial for lithium ion battery.

### 3.2. Electrochemical performance of Co_3_O_4_@CdS nanorod/electrode

#### 3.2.1. Cyclic voltammetry

**Fig. 3:**
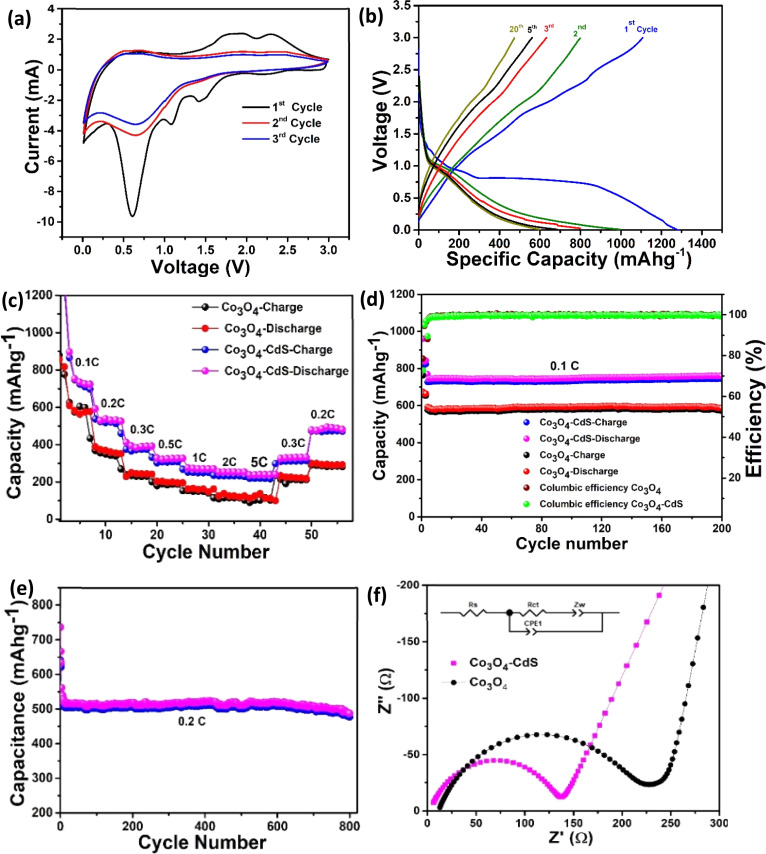
(a) CV curves of the Co_3_O_4_@CdS electrode for 1st, 3rd and 5th cycle at a scan rate of 0.5 mV s^−1^. (b) Galvanostatic charge/discharge profiles of the Co_3_O_4_@CdS electrode (c) rate performance (d) performance of electrodes at 0.1C for 200 cycles and (e) cyclic stability of Co_3_O_4_@CdS electrode at 0.2C for 800 cycles (f) comparison of the EIS curves of Co_3_O_4_@CdS and Co_3_O_4_ electrodes; inset is the kinetic parameters of both electrodes.

The electrochemical performance of the assembled cells is recorded by conducting CV curves. Fig. 3(a) depicts CV curves of Co_3_O_4_@CdS cell for the 1st, 3rd and 5th cycles measured in a voltage range of 0.01–3.0 V (*vs.* Li/Li^+^) at a scan rate of 0.5 mV s^−1^. During the first cycle, cathodic peaks at 1.4, 1.0 and 0.6 V are ascribed to alloying processes, irreversible reactions and formation of solid electrolyte interphase (SEI) layer. In the 1st anodic scan two oxidation peaks at 1.9 and 2.3 V (*vs.* Li/Li^+^) are associated to the multistep dealloying process. In the subsequent cycles, the cathodic peaks at 1.4 and 0.65 V are related to the reduction of cadmium and cobalt to their metallic states while anodic peaks at 1.9 and 2.3 V correspond to the formation of CdS and Co_3_O_4_ and partial decomposition of SEI. The subsequent cycles exhibit no further change in the shape of CV curves. These results show the good reversibility of the Co_3_O_4_@CdS hybrid structure. The CV curves of the Co_3_O_4_ cell for the initial few cycles are also performed under the same condition as shown in Fig. S7 (ESI).†

#### 3.2.2. Galvanostatic charge/discharge

Fig. 3(b) shows the galvanostatic charge/discharge behaviour of Co_3_O_4_@CdS cell for the 1st, 2nd, 5th, 10th and 20th cycles at a rate of 0.1C (1C = 891 mA g^−1^) in the voltage window of 0.01–3.0 V (*versus* Li^+^/Li) at room temperature. As observed, the voltage profiles of the cell show a plateau at 1.2 V and rapidly reaches a plateau at 0.75 V during the first discharge. These plateaus exhibit the conversion of Co_3_O_4_ to CoO (or LixCo_3_O_4_) and Co respectively. The initial charge and discharge capacity values are 1110 and 1292 mA h g^−1^ respectively with a coulombic efficiency of 76%. These capacity values are higher than the theoretical capacity of Co_3_O_4_ (∼891 mA h g^−1^) and also the previously reported electrode materials as shown in Table 1. Moreover, the discharge capacity of the Co_3_O_4_@CdS electrode is found to be 995, 798, 685 and 598 mA h g^−1^ at 0.1C in the subsequent cycles of 2nd, 5th, 10th and 20th respectively. These values are significantly higher than that of the Co_3_O_4_ electrode. In addition, the shape of the charge/discharge cycles remain similar which show the stability of the hybrid structure as anode. For comparison, the voltage profiles of Co_3_O_4_ electrode is also measured as shown in Fig. S8 (ESI).† The initial charge and discharge capacity of Co_3_O_4_ electrode is found to be 505 mA h g^−1^ and 835 mA h g^−1^ respectively. These capacity values are much smaller than Co_3_O_4_@CdS cell. The improved performance of the Co_3_O_4_@CdS electrode may be due to the formation of metal sulfur bond that bridge to accelerate charge transfer between the metal oxide and sulphide.

**Table 1:** Performance comparisons of various Co_3_O_4_ based materials used for the construction of Li-ion batteries

Materials	Synthesis methods	Initial discharge capacities (mA h g^−1^)	Current densities (A g^−1^/C-rate)	Reversible capacity (mA h g^−1^)	Shelf life (cycles)	Ref.
Nanoporous TiO_2_/Co_3_O_4_ composite	One-step dealloying method	998	100 mA g^−1^	295	500	31
Co_3_O_4_ NWs	Decomposition of CoC_2_O_4_·2H_2_O NWs	1027	0.11 A g^−1^	611	50	32
Co_3_O_4_/graphene composite	Facile synthesis	1097	50 A g^−1^	541	30	33
CoTiO_3_/Co_3_O_4_/TiO_2_	Mechanical milling	480	100 mA g^−1^	722	250	34
Co_3_O_4_@TiO_2_ CSNFs	Hydrothermal method	1034	0.2C	632	100	35
PNF Co_3_O_4_	Self-combustion	1108	0.5C	661	100	36
Co_3_O_4_@CNT	Nano-casting method	1260	0.1 A g^−1^	453	30	37
Co_3_O_4_ nanoparticles	Facile synthesis	1105	50 A g^−1^	541	30	33
Co_3_O_4_@C	MOF-derived strategy	1112	250 mA g^−1^	721	500	38
**Co**_**3**_ **O**_**4**_ **@CdS NRs**	**Hydrothermal method**	**1292**	**0.1C**	**760**	**800 (0.2C)**	**This work**

The rate performance of both cells was also investigated. Fig. 3(c) exhibits the comparison of rate performance of Co_3_O_4_ and Co_3_O_4_@CdS cells at current rates ranging between 0.1C to 0.5C. As the current rates increase from 0.1C to 5C, it can be observed that the reversible capacity of the Co_3_O_4_@CdS electrode steadily decreases from 760 mA h g^−1^ to 258 mA h g^−1^. Comparatively, the reversible capacities of the Co_3_O_4_ electrode drop sharply from 620 mA h g^−1^ to 101 mA h g^−1^ at the similar rates (0.1C to 5C). Interestingly, when the current rate retunes at 0.2C, the Co_3_O_4_@CdS electrode restores its original reversible capacity more effectively than the Co_3_O_4_ electrode after 50 cycles. The stability of the electrodes is investigated by testing the cyclic performance of the Co_3_O_4_ and Co_3_O_4_@CdS electrodes over 200 cycles at 0.1C as shown in Fig. 3(d). As observed, both electrodes exhibit good cycling performance. After 200 cycles, the Co_3_O_4_@CdS electrode shows the reversible capacity of 760 mA h g^−1^ with a capacity retention rate of 83.7% and 92.7% respectively. In comparison, the reversible capacity of Co_3_O_4_ electrode is 580 mA h g^−1^ which is much smaller that Co_3_O_4_@CdS electrode. In order to further evaluate the long cyclic stability of the Co_3_O_4_@CdS electrode, galvanostatic charge/discharge are performed for 800 cycles at 0.2C as shown in Fig. 3(e). It can be observed that after 800 cycles, the electrode shows a stable reversible capacity of 520 mA h g^−1^ corresponding to the 90% of the initial capacity. The improved and stable performance of the Co_3_O_4_@CdS electrode is associated with its mesoporous nature which provides more active sites for Li^+^ insertion/extraction process. These findings demonstrate that the Co_3_O_4_@CdS structure is a suitable choice for the construction of high-rate performance batteries.

#### 3.2.3. Electrochemical impedance spectroscopy

To investigate the kinetic behaviours and charge transfer resistance of the electrodes, EIS study is performed. Fig. 3(f) present the comparative Nyquist plots of the Co_3_O_4_@CdS and Co_3_O_4_ electrodes. It can be observed, in the high frequency region, the plot of Co_3_O_4_@CdS electrode exhibits small diameter of the semicircle as compare to Co_3_O_4_ electrode. This illustrate a low charge transfer resistance (135.1 Ω) and fast transport during the electrochemical process between electrode material and electrolyte. To find the kinetic parameters, the experimental results are fitted well by employing the equivalent circuit model as shown in the inset. The fitting parameters of the two electrodes are presented in Table 2, which shows the excellent performance of the Co_3_O_4_@CdS electrode.

**Table 2:** Comparison of kinetic parameters of Co_3_O_4_@CdS and Co_3_O_4_ electrodes

Material	*R*_s_ (Ω)	*R*_ct_ (Ω)
Co_3_O_4_@CdS	2.615	135.1
Co_3_O_4_	10.88	223.6

### 3.3. DFT calculations

**Fig. 4:**
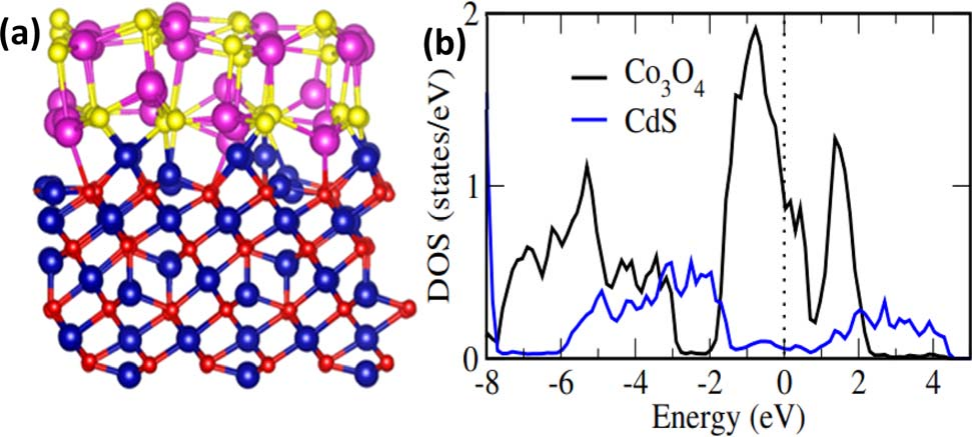
(a) Schematic description of the optimized Co_3_O_4_ (111)/CdS (002) interface. Colours; blue (Co), red (O), purple (Cd) and yellow (S), (b) density of states (DOS) contributions of Co_3_O_4_ and CdS surfaces within Co_3_O_4_@CdS hybrid structure. DOS is normalized to the total number of atoms within each surface to provide a clear comparison. Dashed vertical line presents Fermi level (*E*_F_).

To further understand the experimental findings, DFT calculations is performed. The optimized structure for Co_3_O_4_ (111)/CdS (002) interface is shown in Fig. 4(a). Evidently, there is a strong interaction at the interface between Co_3_O_4_ and CdS (002) atoms, dominated by Co–S and Cd–S bonds. The average Co_3_O_4_ (111)–CdS (002) separation is ∼2.30 Å suggesting the presence of chemical bonding besides weak vdW interactions. Besides covalent bonding, interfacial interactions also have ionic character; Bader analysis^28^ indicates that there is charge transfer of ∼0.65*e* from Co_3_O_4_ to CdS. These findings are consistent with the recent experimental work where both the presence of Co–S interfacial bonds as well as an increase (reduction) in electronic density was observed for CdS (Co_3_O_4_) surface within Co_3_O_4_@CdS.^29^

The impact of strong interfacial interactions on electronic structure is highlighted in Fig. 4(b) where density of states (DOS) contributions of Co_3_O_4_ and CdS within Co_3_O_4_@CdS are presented. Interestingly, CdS has states around Fermi level (*E*_F_) suggesting a metallic behaviour. This is in contrast to pristine CdS layer which has a large band gap of ∼2.0 eV Fig. S9 (ESI).† Therefore, it is evident that transport character of CdS layer on Co_3_O_4_ changes from semiconducting to metallic, owing to strong interfacial interactions. Moreover, conductivity of Co_3_O_4_@CdS will be higher as compared to the individual pristine surfaces, especially close to the interface region. Overall, both the increase in conductivity of Co_3_O_4_@CdS as well as higher electronic density of CdS due to interfacial charge transfer will improve the reaction kinetics at the electrode.^30^ This is indeed observed in the impedance spectroscopy (Table 2) where a noticeable reduction in charge transfer resistance (*R*_ct_) is observed for Co_3_O_4_@CdS in comparison to that of pristine Co_3_O_4_.

The improved lithium storage performance of the Co_3_O_4_@CdS hybrid structure may have following possible reasons (i) the functionalization of CdS NPs increase the surface reactivity and porosity which creates abundant active sites/interfaces for the rapid diffusion and transportation of Li^+^ ions during the electrochemical reaction (ii) the addition of CdS NPs increase the contact area between electrolyte and electrode and protect the Co_3_O_4_ nanosheets from degradation during the charge and discharges process. (iii) The synergy between Co_3_O_4_ and CdS NPs greatly improved the kinetics of Co_3_O_4_@CdS nanosheets for fast lithium storage.

## 4 Conclusion

In summary, a novel mesoporous Co_3_O_4_@CdS hybrid structure were successfully synthesized by employing hydrothermal along with SILAR method. The hybrid structure demonstrates several structural features including the large specific surface area, abundant active sites and excellent kinetics. The developed electrode showed improved lithium storage performance as compare with pristine and previously reported electrodes. The electrode delivers a stable reversible capacity of 760 mA h g^−1^ at 0.1C over 200 cycles with a capacity retention rate of 92.7%. The electrode can achieve reversible capacity of 520 mA h g^−1^ at 0.2C even after 800 cycles. The improved lithium storage performance may be due to the increase in kinetics, synergy and abundant active sites that facilitate the storing of more Li^+^ ions and fast transportation during the lithiation/delithiation. It is suggested that the electrochemical performance of Co_3_O_4_@CdS hybrid structure can be further enhanced by structural engineering and optimizing the amount of CdS NPs. This work provides a novel platform to construct a LIBs with long cyclic stability and high-rate capability.

## Author contributions

M. A. conceived the idea and designed the experiment. H. W. and H. R. synthesized materials. S. K., F. F and A. N. analyzed the XRD and FESEM data. Y. Y., A. K. and H. S. carried out the HRTEM characterization and analysis. S. J. carried out the DFT calculations. H. W., M. A., and A. K. performed coin cell measurements, analyzed the XPS data. S. H. performed the Raman measurements. A. Z. conducted the electrochemical impedance spectroscopic measurements. H. W. and M. A. performed the CV and GCD measurements. H. W., M. A., and A. N. co-wrote the paper. All authors discussed the results and commented on the manuscript. M. A. and A. N. supervised the whole research work.

## Conflicts of interest

The authors declare no conflict of interest.

## Supplementary Material

RA-014-D4RA01028K-s001
