# Supplementary material for: Mesoporous Co3O4@CdS nanorods as anode for high-performance lithium ion batteries with improved lithium storage capacity and cycle life
Source: RSC Adv. 2024 Apr 15;14(17):11900–7. doi: 10.1039/d4ra01028k (PMC11017193; doi:10.1039/d4ra01028k)
Supplement: RA-014-D4RA01028K-s001 [file RA-014-D4RA01028K-s001.pdf]

Electronic Supplementary Material (ESI) for RSC Advances.
This journal is © The Royal Society of Chemistry 2024

Electronic Supplementary Information (ESI)

Mesoporous Co_3O_4@CdS Nanorods as anode for high-performance lithium ion batteries with improved lithium storage capacity and cycle life

Hamza Waleed [a, i], Haroon Ur Rasheed [a, i], Faisal Faiz [b], Amina Zafar [c], Saqib Javed [d], Yanguo Liu [e], Shafqat Karim [a], Hongyu Sun [e], Yasir Faiz [f], Shafqat Hussain [a], Atia Khalid [g], Yanlong Yu [*,h], Amjad Nisar [*,a] and Mashkoor Ahmad [*,a]

a. *Nanomaterials Research Group, Physics Division, PINSTECH, Islamabad 44000, Pakistan.*

b. *State Key Laboratory of Analytical Chemistry for Life Science, School of Chemistry & Chemical Engineering and Center of Materials Analysis, Nanjing University, Nanjing 210023, China.*

c. *Central Analytical Facility Division, PINSTECH, Islamabad 44000, Pakistan.*

d. *Theoretical Physics Division, PINSTECH, Islamabad 44000, Pakistan.*

e. *School of Resources and Materials, Northeastern University at Qinhuangdao, Qinhuangdao, 066004, PR China.*

f. *Chemistry Division, PINSTECH, Islamabad 44000, Pakistan.*

g. *School of Materials Science and Engineering, Tsinghua University, Beijing China.*

h. *College of Chemistry and Chemical Engineering, Northeast Petroleum University, Daqing, 163318, PR China.*

i. *Faculty of Basic & Applied Sciences, Department of Physics, IIU, Islamabad, 44000, Pakistan.*

Correspondence:
mashkoorahmad2003@yahoo.com (Mashkoor Ahmad)
chempk@gmail.com (Amjad Nisar)
ylyu66@163.com (Yanlong Yu)

2. Experimental

2.1. Reagents and materials

Cobalt nitrate hexahydrate Co $(NO_3)_2.6H_2O$, and hexamethylenetetramine (HMT) were purchased from Scharlau. Cadmium chloride hexahydrate ($CdCl_2.6H_2O$) and sodium sulfide hydrate ($Na_2S.H_2O$) are supplied by Sigma Aldrich. Deionized (DI) water was used throughout the experiment. All the reagents were purchased and used without any further purification and available at commercial scale.

2.2. Synthesis of Co_3O_4 nanorods

Cobalt oxide (Co_3O_4) was prepared via facile hydrothermal method. In this process, 1.4g cobalt nitrate hexahydrate (Co $(NO_3)_2.6H_2O$) and 1.45g hexamethylenetetramine (HMT) were added in 40 ml deionized water and stirred for 30 minutes. The prepared homogeneous solution was added into a Teflon-lined stainless-steel autoclave (with the capacity of 60 ml). The autoclave was placed in an oven for 6 hours at 95 ° C. Pink-colored cobalt oxide (Co_3O_4) powder was obtained after the completion of the reaction. The product was obtained after centrifugation and then washed continuously with distilled (DI) water and ethanol to avoid impurities. The obtained products were dried in an oven at 60 °C for 5 hours. Finally, the products were annealed for 2 hours at 450° C. After annealing, the colour of the product was changed into black.

2.3. Synthesis of Co_3O_4@CdS hybrid structure

The synthesis of Co_3O_4@CdS hybrid structure was carried out by employing Successive Ion Layer Adsorption and Reaction (SILAR) method for homogenous and uniform deposition of CdS nanoparticles on Co_3O_4 nanorods. In this process, four different glass beakers were arranged in a sequence, two of them were filled with 0.05 M $CdCl_2.6H_2O$ and 0.05 M $Na_2S.H_2O$ aqueous solution and the other with DI water. The SILAR cycle was proceeded as: firstly, the

annealed Co_3O_4 nanorods was sonicated in $CdCl_2.6H_2O$ solution for 5 min, secondly, into distilled water, thirdly, into $Na_2S.H_2O$ solution, and lastly, washed with DI water. The prepared material was then dried at room temperature for 5 hours.

2.4. Characterization

The morphology and elemental composition of the samples were examined by field emission scanning electron microscopy (FESEM, TESCAN MIRA-3) furnished with energy dispersive spectroscopy (EDS). The phase purity was carried out by using a Bruker Model D8 Advance X-ray powder diffractometer (XRD) with X-rays of Cu-Kα source (λ=1.5418Å). Fourier Transform Infrared (FTIR) spectroscopy was performed by a Nicolet iS50 FT-IR spectrometer. The detailed structural analysis was analysed by high resolution transmission electron microscopy (HR-TEM, JEOL-JEM-201, 200kV). The Raman spectroscopy was carried out by using a Horbia Xplora spectrometer with laser wavelength 532 nm. XPS was performed by using Super ESCA beam at Electra, Trieste, Italy.

2.5. Coin Cell Assembly and measurements

The working electrode was prepared by dispersing active material, carbon black and soft binder PVDF (polyvinylidene fluoride) in NMP (N-methylpyrrolidone) solvent with a weight ratio of 70:20:10. The slurry was spread on copper foil and dried in a vacuum oven at 60 °C overnight then cut into disks (diameter 15 mm). The cells were assembled in an Ar-filled glovebox using lithium foil as negative electrode, porous polypropylene as the separator, and 1M LiPF6 in ethylene carbonate, dimethyl carbonate and ethylene methyl carbonate (2:1:2 by volume) was used as the electrolyte. The electrochemical performance of the cells were performed by testing galvanostatic charge/discharge at various current densities using 8 Channel Battery Analyzer system (BTS8) in the voltage range of 0.01-3.0 V (vs.Li/Li+). Cyclic voltammogram (CV; 0.01-3.0 V, 5 mVs^{-1}) measurements were carried out using a CHI660C (Chenghua, Shanghai)

electrochemical workstation. The electrochemical impedance spectroscopy (EIS) measurements were recored using CHI660C workstation with amplitude of 5 mV in the frequency range of 100 kHz to 0.01Hz.

2.6. Computational Details

DFT calculations are performed by employing plane wave pseudo potential method as implemented in VASP code [12]. Generalized gradient approximation within Perdew–Burke–Ernzerhof (PBE) parameterizationis used as an exchange correlation functional [13]. Van der Waals(vdW) dispersion corrections are also included by using DFT-D3 approach [14].

To describe Co_3O_4@CdS hybrid structure, Co_3O_4 (111) and CdS (002) surfaces are selected which also observed in the TEM results **(Figure 1)**; both surfaces have similar planar structure which should facilitate the formation of a coherent interface. Specifically, the interface comprised of 12 layers of Co_3O_4 (111) and 4 layers CdS (002). Further, in plane lattice parameters of Co_3O_4 (111) and CdS (002) were expanded 3×3 and 4×4 times, respectively to ensure that the lattice mismatch is within acceptable limit (< 5 %). A vacuum spacing of ~ 15 Å along z-axis was provided to mitigate spurious interaction between periodic images. Kinetic energy cut off of 500 eV was used to ensure the convergence of the results. A single Γ-point was used for Brillion zone sampling while convergence criteria for total energy and forces was set to 10^{-5} eV and 0.02 eV/Å, respectively.

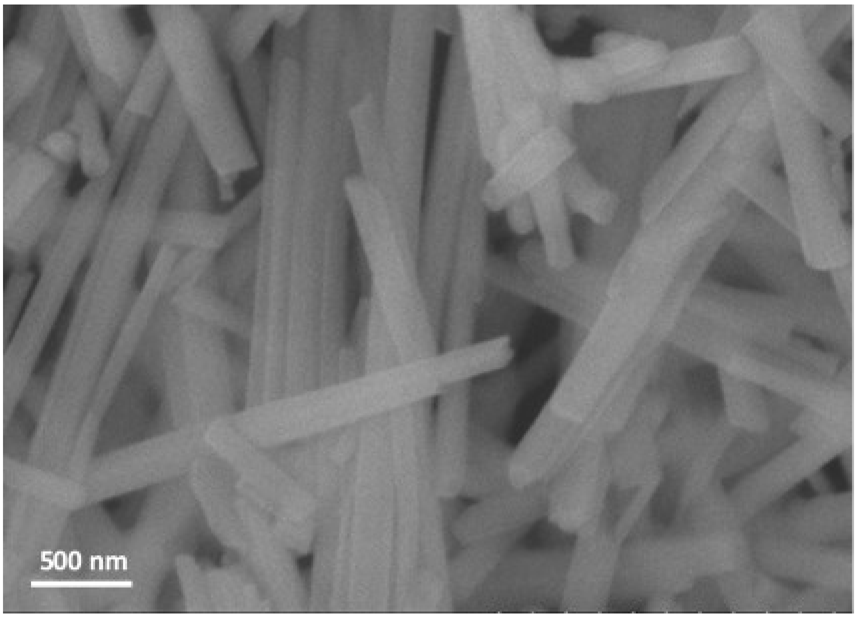

Figure S1. SEM images of pristine Co_3O_4 nanorods

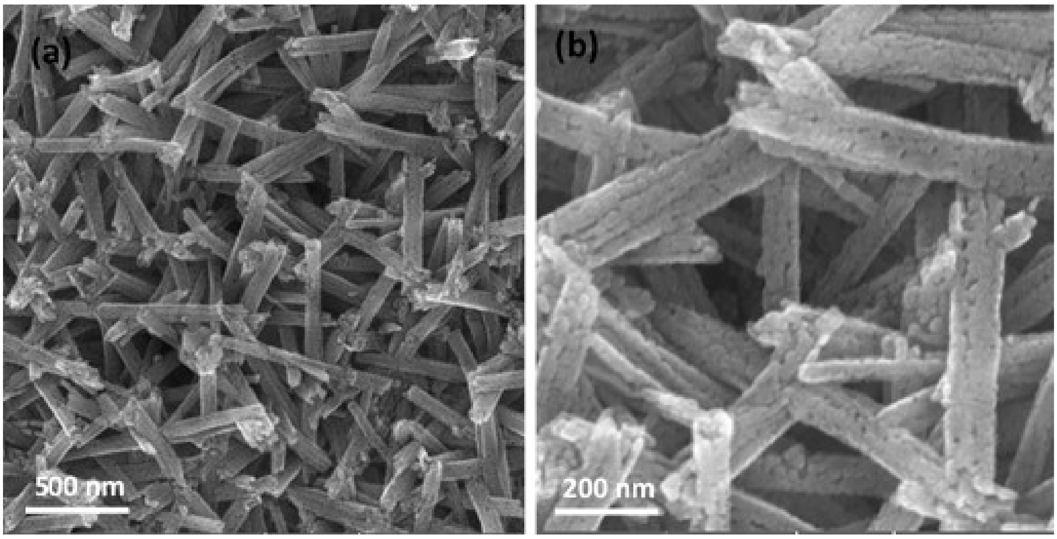

Figure S2. SEM images of Co_3O_4@CdS nanorods

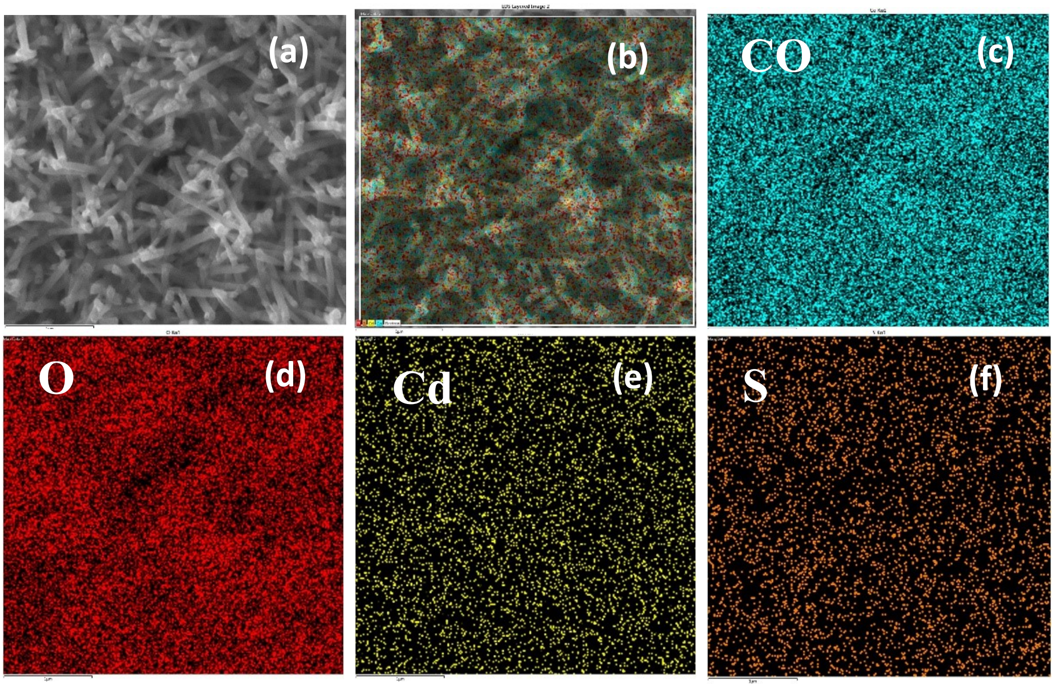

Figure S3. (a, b) SEM images of Co_3O_4@CdS nanorods (c-f) EDX elemental mapping analysis for Co, O, Cd and S in Co_3O_4@CdS nanorods

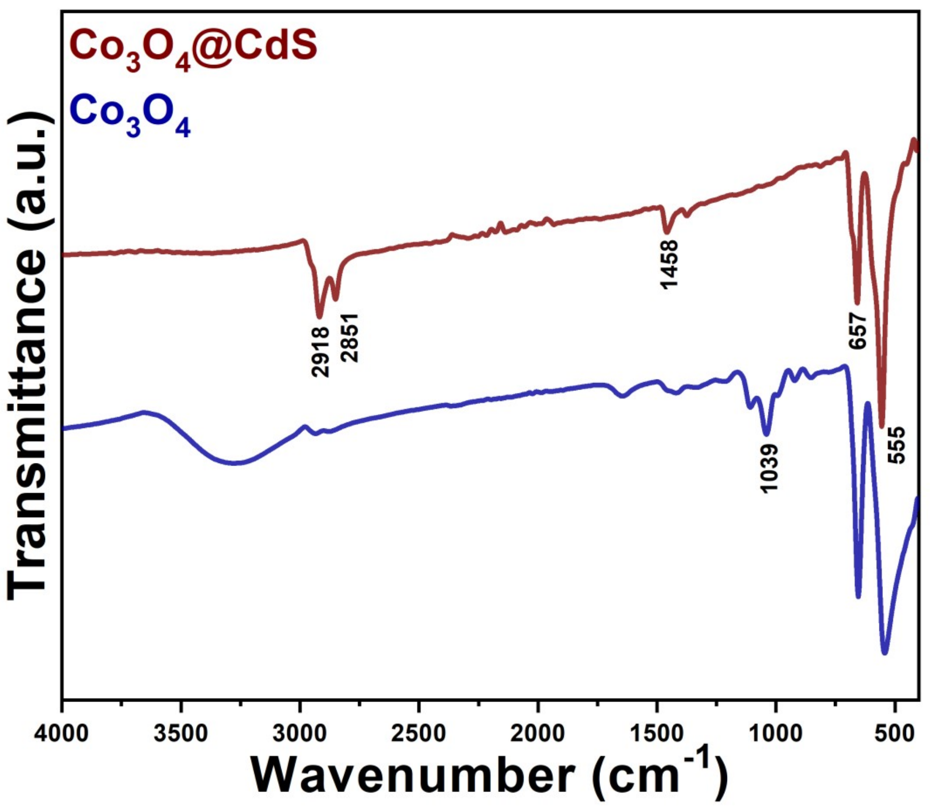

Figure S4. FTIR spectra of Co_3O_4 and Co_3O_4@CdS structures prepared by modified SILAR method.

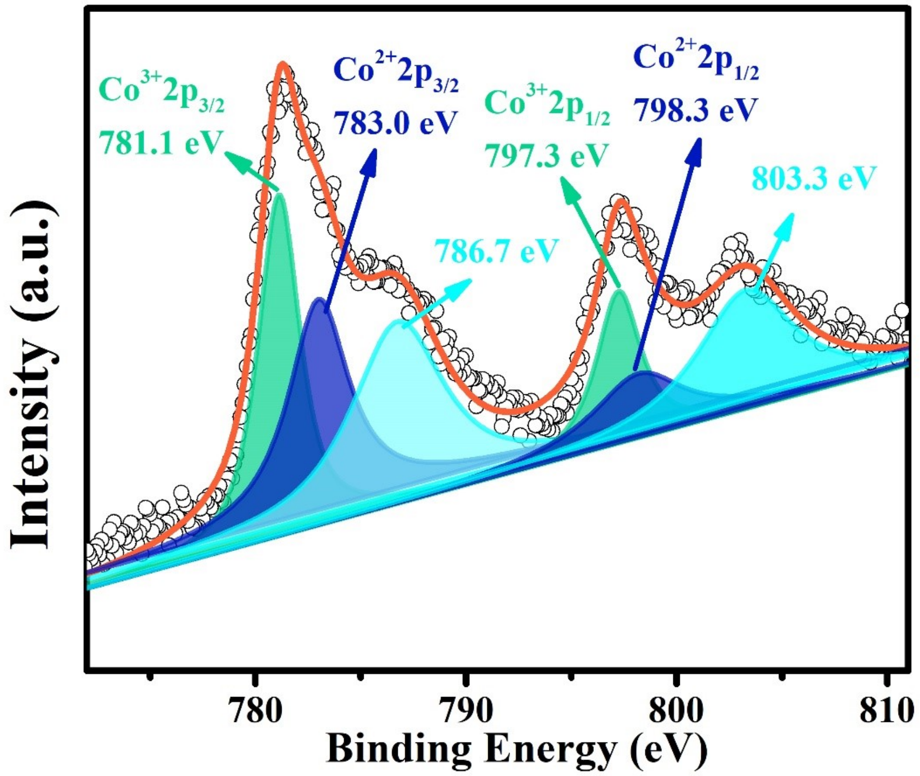

Figure S5. High resolution XPS spectrum of Co 2p peak of Co_3O_4 nanorods.

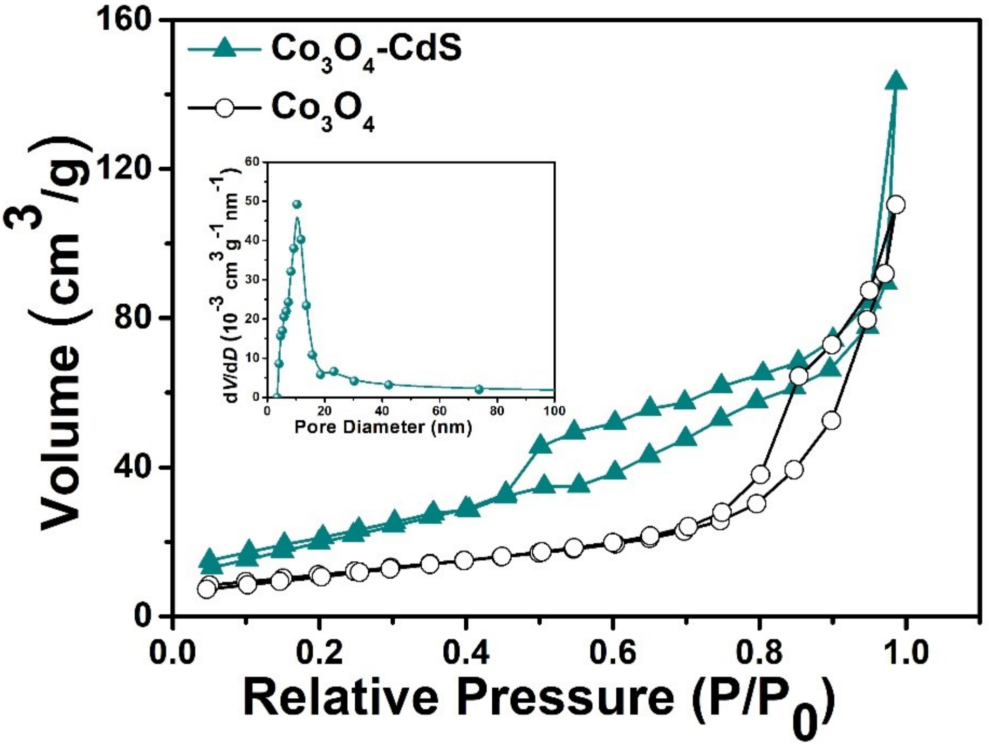

Figure S6. Nitrogen adsorption–desorption isotherms for pristine Co_3O_4 and Co_3O_4@CdS structures; inset demonstrates the pore size distribution of Co_3O_4@CdS structure.

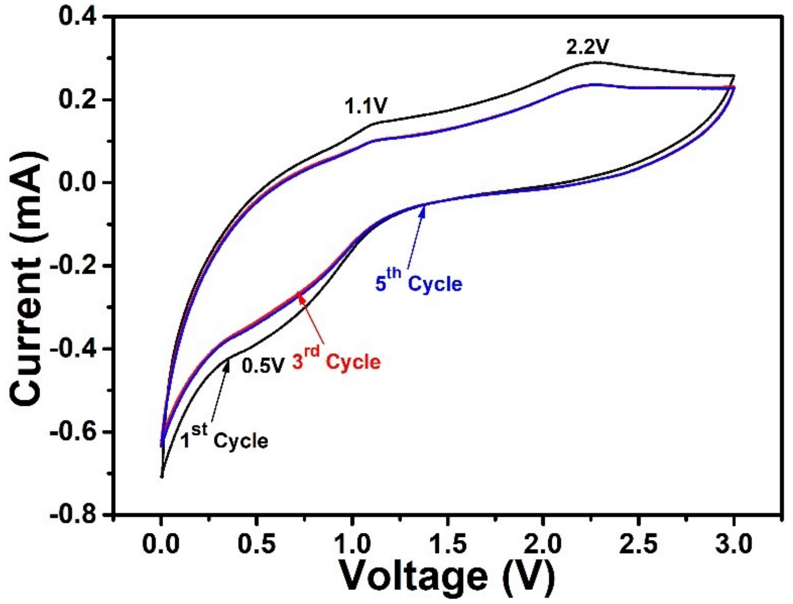

Figure S7. CV curves of the Co_3O_4 electrode for 1st, 3rd and 5th cycle at a scan rate of 0.5 mVs^{-1}.

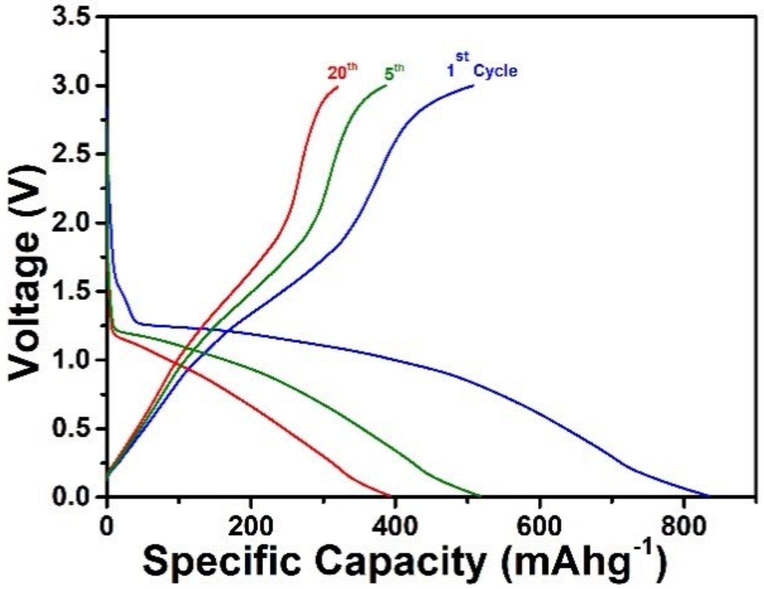

Figure S8. Galvanostatic charge/discharge profiles of pristine Co_3O_4 electrode.

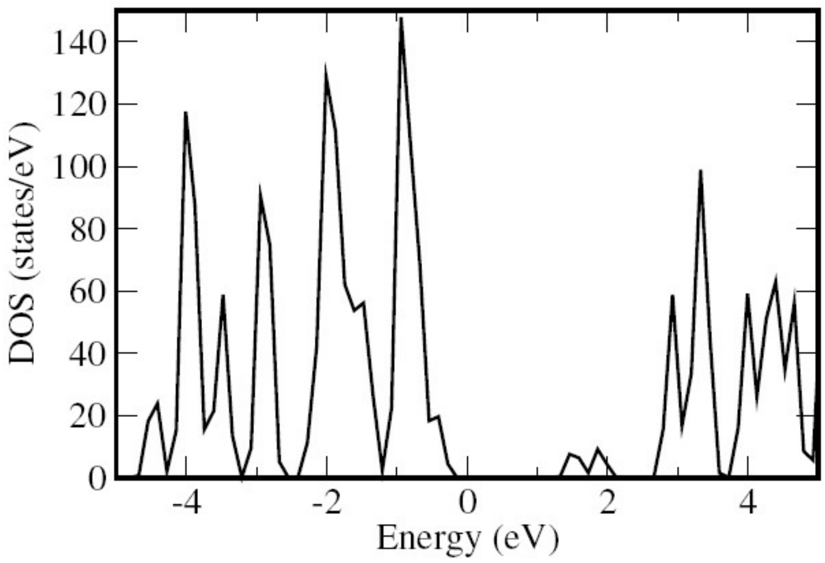

Figure S9. Density of states (DOS) of pristine CdS (002) surfaces. Zero of abscissa denotes Fermi level (E_F).
